# Expanded genetic testing of GIST patients identifies high proportion of non-syndromic patients with germline alterations

**DOI:** 10.1038/s41698-022-00342-z

**Published:** 2023-01-02

**Authors:** Diana Mandelker, Antonio Marra, Nikita Mehta, Pier Selenica, Zarina Yelskaya, Ciyu Yang, Joshua Somar, Miika Mehine, Maksym Misyura, Olca Basturk, Alicia Latham, Maria Carlo, Michael Walsh, Zsofia K. Stadler, Kenneth Offit, Chaitanya Bandlamudi, Meera Hameed, Ping Chi, Jorge S. Reis-Filho, Ozge Ceyhan-Birsoy

**Affiliations:** 1 Department of Pathology and Laboratory Medicine, Memorial Sloan Kettering Cancer Center, New York, NY USA; 2 Marie-Josee and Henry R. Kravis Center for Molecular Oncology, Memorial Sloan Kettering Cancer Center, New York, NY USA; 3 Department of Medicine, Memorial Sloan Kettering Cancer Center, New York, NY USA

**Keywords:** Molecular medicine, Cancer genetics, Cancer genomics

## Abstract

Traditional genetic testing for patients with gastrointestinal stromal tumors (GISTs) focus on those with syndromic features. To assess whether expanded genetic testing of GIST patients could identify hereditary cancer predisposition, we analyzed matched tumor-germline sequencing results from 103 patients with GISTs over a 6-year period. Germline pathogenic/likely pathogenic (P/LP) variants in GIST-associated genes (*SDHA, SDHB, SDHC, NF1, KIT*) were identified in 69% of patients with *KIT/PDGFRA*-wildtype GISTs, 63% of whom did not have any personal or family history of syndromic features. To evaluate the frequency of somatic versus germline variants identified in tumor-only sequencing of GISTs, we analyzed 499 de-identified tumor-normal pairs. P/LP variants in certain genes (e.g., *BRCA1/2, SDHB*) identified in tumor-only sequencing of GISTs were almost exclusively germline in origin. Our results provide guidance for genetic testing of GIST patients and indicate that germline testing should be offered to all patients with *KIT/PDGFRA*-wildtype GISTs regardless of their history of syndromic features.

Gastrointestinal stromal tumors (GISTs) are the most common mesenchymal tumors of the gastrointestinal system^1^. Up to 85–90% of GISTs harbor activating mutations in *KIT* or *PDGFRA* and respond to imatinib or sunitinib therapies^2,3^. The remaining 10–15% are *KIT/PDGFRA*-wildtype and may have somatic loss-of-function (LOF) mutations in succinate dehydrogenase (SDH)-complex subunits (*SDHA/B/C/D*) or *NF1*, activating mutations in *BRAF*, *FGFR1*, or *NTRK* translocations, or may be driven by germline alterations^1,3–5^. Germline pathogenic variants in SDH-complex genes^6–8^, *NF1*^9^, *KIT*^10,11^, and *PDGFRA*^12,13^ have been associated with the development of GISTs. Currently, no standard guidelines exist for germline testing in GIST patients. Previous studies on germline contribution to GISTs focused on patients with *KIT/PDGFRA*-wildtype tumors, particularly those with syndromic features, and targeted selected genes^4,5,7^, given the assumption that GISTs resulting from germline alterations are frequently syndromic^3,4^. Recent studies, however, demonstrated that syndromic features may be absent in a large proportion of patients with hereditary cancer predisposition^1,14,15^.

Owing to the restrictive pre-selection of patients and genes for genetic testing, the contribution of germline variants to GIST development remains to be fully characterized. To assess whether expanded genetic testing of unselected GIST patients could identify individuals with hereditary predisposition, we analyzed matched tumor-germline sequencing results from 103 patients with GISTs treated at Memorial Sloan Kettering (MSK) Cancer Center (MSKCC) over a 6-year period (Supplementary Fig. 1). Testing was performed using MSK Integrated Mutation Profiling of Actionable Cancer Targets (MSK-IMPACT)^16^ and included 76–90 cancer predisposition genes (Supplementary Table 1).

Overall, of the 103 patients with GISTs in this cohort, 24 (23%) had a germline pathogenic/likely pathogenic (P/LP) variant in a GIST-associated gene. The cohort of 103 patients included 58 with somatic mutations in *KIT*, 10 with somatic mutations in *PDGFRA*, and 35 with *KIT/PDGFRA*-wildtype GISTs (Fig. 1, Supplementary Table 2). Strikingly, 69% (24/35) of individuals with a *KIT/PDGFRA*-wildtype GIST harbored a germline P/LP variant in a GIST-associated gene (Fig. 2a, Supplementary Fig. 1). These included 16 patients with SDH-complex gene (seven *SDHA*, seven *SDHB*, two *SDHC*), seven with *NF1*, and one with *KIT* germline P/LP variants, accounting for 46%, 20%, and 3% of patients with *KIT/PDGFRA*-wildtype GISTs, respectively. Seven (20%) of the patients with *KIT/PDGFRA*-wildtype GISTs had other somatic driver mutations: four with *NF1* biallelic, one with *SDHA* biallelic LOF, and two with *BRAF* activating mutations. Three tumors from patients without germline P/LP variants had monoallelic *SDHB* or *SDHD* mutations, and due to the limitations in detecting LOH or a second mutation, were considered as inconclusive for somatic mutation status. One patient had neither germline nor somatic mutations identified in GIST-associated genes. *SDHC* promoter methylation^17^ was tested and ruled out in tumors without conclusive driver mutations. Overall, germline P/LP variants in GIST-associated genes were identified in 96% (24/25) of patients with somatic driver-negative tumors.

**Fig. 1:**
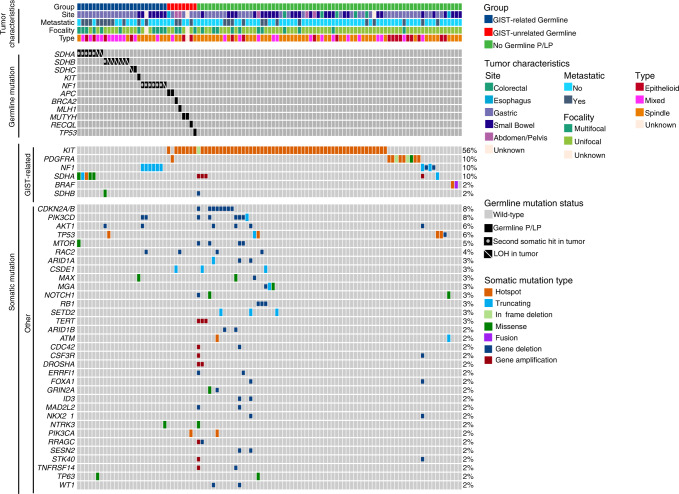
Mutational landscape of GISTs from 103 patients who received germline genetic testing. Tumor characteristics (site, metastasis status, focality, type) and the effects of somatic mutations are color coded according to the legend. LOH is displayed by a diagonal bar and second somatic mutation (second somatic hit) is displayed by a dot. The numbers on the right side of each row refer to the percentage of tumors with that given gene mutation in the overall cohort of 103 patients.

**Fig. 2:**
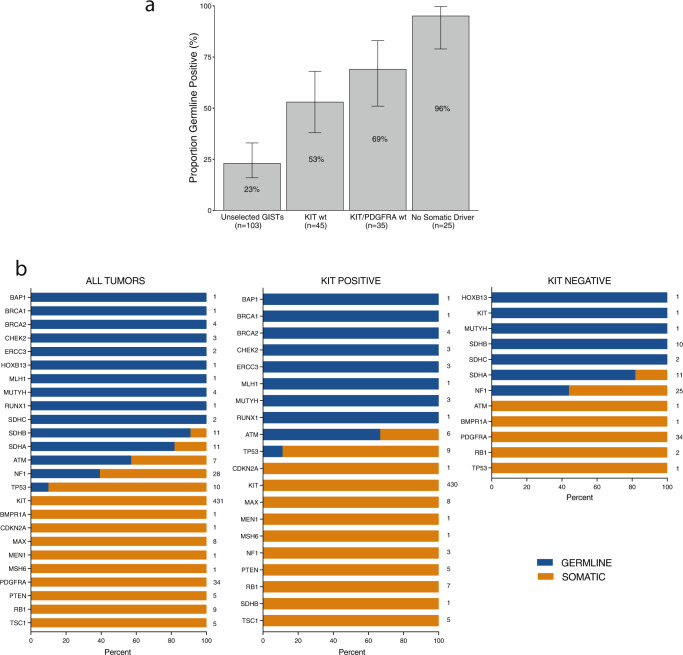
Detection of germline P/LP variants in GIST patients based on somatic profiles. **a** Proportion of patients with P/LP variants in GIST-associated genes in the cohort of 103 patients who received germline genetic testing. Percentages are plotted and displayed on the bar graphs. Error bars represent confidence intervals calculated by the Clopper-Pearson method. **b** Distribution of germline (blue) and somatic (orange) P/LP variants detected in tumor-only sequencing analysis of de-identified GISTs from 499 patients. Data from all tumors (left), *KIT*-mutant tumors (middle), and *KIT*-wildtype tumors (right) are presented. Numbers displayed on the right of each gene data on each plot represent the number of tumor samples with variants identified in each gene in that group of tumors.

Germline P/LP variants in cancer predisposition genes not known to be associated with GISTs were identified in eight patients, all of whom had *KIT/PDGFRA*-mutant GISTs. These included alterations affecting *TP53, MLH1, BRCA2*, and *RECQL* in one patient each, as well as two patients with *APC* p.Ile1307Lys low-penetrance variant and two with monoallelic *MUTYH* variants (Supplementary Table 2). Loss-of-heterozygosity (LOH) or a second somatic mutation in these genes were not observed in the tumors. The patient with *MLH1* alteration had a microsatellite-stable tumor and homologous recombination deficiency signature was not detected in the patient with *BRCA2* alteration, suggesting that these variants likely constitute incidental findings.

Patients with germline GIST-associated variants had a younger median age-of-onset (39.5 vs 52 years, *p* = 0.01), and were more likely to have metastatic disease (50% vs 20%; *p* = 0.01) and multifocal lesions (50% vs 18%, *p* = 0.01) at the time of testing compared to those lacking P/LP variants in these genes (Supplementary Table 3).

Most patients with germline P/LP variants in GIST-associated genes did not present with syndromic features. Of the 16 patients with germline SDH-complex gene defects, only one had syndromic presentation (history of paraganglioma), and another had family history (father with paraganglioma), whereas 88% (14/16) had no personal or family history of syndromic features at the time of testing. Thirteen patients had SDHB immunohistochemistry performed, and tumors demonstrated absence of SDHB expression. While 86% (6/7) of patients with germline *NF1* alterations had a history of NF1 features, for one patient, identification of the germline *NF1* variant led to the recognition of café-au-lait spots and mild axillary/inguinal freckling, consistent with mild neurofibromatosis^15^. Additionally, one (1%) of 103 patients had a pathogenic germline *KIT* variant (p.Lys509Ile)^18,19^. Overall, 63% (15/24) of patients with germline GIST-associated variants did not have personal or family history of syndromic features, suggesting that a significant proportion of *KIT/PDGFRA*-wildtype GISTs appearing to be sporadic may have underlying germline alterations.

Tumor-only sequencing is commonly performed to assess somatic alterations and may reveal germline variants. To determine the frequency of somatic versus germline variants identified in tumor-only sequencing of GISTs, we analyzed a cohort of de-identified 499 GISTs that received paired tumor-normal sequencing using MSK-IMPACT, including tumors from the 103 patients in the initial analysis and 396 patients who did not consent to germline testing (Supplementary Fig. 1). To mimic a tumor-only sequencing analysis approach, the sequencing data from these tumors was used in an unmatched manner, without subtracting the variant calls detected in the paired normal sequencing data. For distinguishing germline versus somatic variants in the tumor sequencing data, variant allele fraction (VAF) thresholds for predicting germline variants in tumor sequencing recommended by the European Society of Medical Oncology (VAF of >30% for single nucleotide variants (SNVs) and >20% for insertions and deletions (indels))^20^ were applied to P/LP variants identified in the tumors. The matched normal blood sequencing data was used to confirm the germline versus somatic origin of the variants detected in tumors. These VAF thresholds correctly distinguished all true germline and somatic P/LP variants identified in the tumors, as determined by comparison with the matched normal blood sequencing data. Of the 69 *KIT/PDGFRA*-wildtype GISTs in this cohort, 33 (48%) were found to harbor germline P/LP variants in GIST-associated genes, consistent with our findings in the cohort who received germline testing. P/LP variants identified in tumor-only sequencing of GISTs were almost exclusively germline in certain genes, such as *BRCA1*, *BRCA2* (4/4 variants), and *SDHB* (10/11 variants). Conversely, P/LP variants in genes such as *KIT* (430/431 variants) and *RB1* (9/9 variants) were primarily somatic (Fig. 2b). These observations can help identify the subset of GIST patients to offer germline testing based on tumor-only sequencing results.

This study has limitations. Detailed clinical assessments could only be performed for 103 patients who consented to germline testing. Additionally, limitations exist in detecting certain variants such as structural variants or low-level mosaicism.

Despite these limitations, our results support that germline testing should be offered to all patients with *KIT/PDGFRA*-wildtype GISTs, regardless of their history of syndromic features, and should target all GIST-associated genes at minimum. In our cohort of 103 patients with GISTs who consented to germline analysis, 23% had a germline P/LP variant in a GIST-associated gene, and an additional 8% had a germline P/LP variant in an additional cancer susceptibility gene, suggesting that all GIST patients, regardless of their tumor testing status, could benefit from germline testing. Germline alterations in GIST-associated genes may also confer high risk for other cancers, such as paragangliomas and pheochromocytomas for SDH-complex genes and nerve sheath tumors, breast cancer, and gliomas for *NF1*. Therefore, their identification has critical implications for future cancer surveillance and clinical management of the patients, as well as appropriate care of their at-risk family members. Determining the somatic and germline alterations underlining tumor development is also critical for appropriate targeted therapy selections, as SDH-deficient and *NF1*-related tumors are known to respond poorly to traditional imatinib therapy and differentiating the different genetic alterations in *KIT/PDGFRA*-wildtype GISTs can guide treatment choices^3,4^. Finally, our analysis on 499 tumor-normal pairs suggests that the identification of P/LP variants in certain genes in tumor-only sequencing of GISTs may indicate germline testing. Additional large-scale studies on the phenotypic spectrum of GIST-associated genes will help in developing clinical guidelines for expanded genetic testing in GIST patients.

## Methods

### Patient cohort

The de-identified cohort consisted of tumor-normal pairs from 499 consecutive patients with GIST who were treated at MSKCC and had MSK-IMPACT (ClinicalTrials.gov identifier, NCT01775072) paired tumor-blood DNA sequencing test^21,22^ between April 2015 and June 2021. Germline analysis cohort consisted of 103 patients with GIST, who were a subset of the larger cohort and prospectively consented to germline analysis as part of MSK-IMPACT. Patients were ascertained through their treating physicians and referral was at the discretion of the physicians. The presence/absence of personal/family history of syndromic features were determined based on review of pre-testing and post-testing clinical geneticist and oncologist physical examination and family history assessments that included inquiry about features related to genetic disorders associated with the development of GISTs. All patients provided written informed consent for testing under a Memorial Sloan Kettering Cancer Center Institutional Review Board (IRB)-approved protocol (IRB#12-245).

### MSK-IMPACT testing

MSK-IMPACT is a New York State Department of Health approved assay and was performed in our CLIA-approved laboratory. Next-generation sequencing was performed on DNA isolated from matched blood and tumor specimens, as described^16,21–23^. DNA fragments were captured using custom-designed biotinylated probes (NimbleGen) and sequenced on an Illumina HiSeq 2500 as paired-end 100 bp reads^21,22^. Tumor testing included 341, 410, or 505 genes, depending on the panel used for testing. Germline testing included 76, 88, or 90 hereditary cancer predisposition genes for 18, 71, and 14 patients, respectively (Supplementary Table 1)^16,22^. Variants were called using MuTect^24^ and Genome Analysis Toolkit (GATK) Haplotype caller^25^. Copy number variants (deletions and duplications of single or multiple exons) were detected and analyzed using an in-house developed pipeline^16,22,23^, which identifies copy number aberrations by comparing sequence coverage of targeted regions to standard diploid normal. Variants were filtered using 25% (for SNVs) and 15% (for indels) variant allele fraction and 20× coverage thresholds. All variants with <1% population frequency in the Genome Aggregation Database (gnomAD)^26^ were reviewed and classified by clinical molecular geneticists and molecular genetic pathologists based on the American College of Medical Genetics and Genomics (ACMG) guidelines^27^.

### LOH, microsatellite instability (MSI), and mutation signature analyses

LOH was determined using segmented allele-specific copy number calls from FACETS tool^28^, an open-source software that utilizes aligned sequence bam files from next-generation sequencing and performs analysis for joint segmentation of total- and allele-specific read counts and integer copy number calls corrected for tumor purity, ploidy and clonal heterogeneity to estimate LOH. Segments with a minor allele copy number of 0 were classified as having LOH. The allele undergoing LOH was determined based on the VAF of the germline variants in the tumor. MSI status of the tumors were assessed using clinically validated MSIsensor program^29^, which computes length distributions of microsatellites per designated regions in paired tumor and normal sequence data and identifies the percentage of microsatellite loci that are unstable in the tumor genome as compared to its matched normal. Evidence of MSI at ≥10% of analyzed loci was considered as MSI-high, ≥3 to <10% was considered as indeterminate MSI status, and <3% were considered as microsatellite stable. Tumor mutation signatures were determined based on MSK-IMPACT data by assigning the mutations in each sample to constituent mutation signatures from a set of 30 signatures described previously^23,30,31^.

### *SDHC* promoter methylation testing

DNA isolated from tumor tissues were subjected to bisulfite treatment, followed by PCR and PyroSequencing, and *SDHC* promoter methylation status was evaluated as described^17^. Samples were run with in-house unmethylated DNA as negative control and CpGenome Human Methylated DNA standard (EMD Millipore Corp., MA, USA) as positive control. Genomic DNA was treated with bisulfite conversion using EpiTect Bisulfite Kit (Qiagen, Hilden, Germany). Each PCR amplification reaction of bisulfite-treated DNA was performed in 50 µl reaction consisting of 35 µl SIGMA JumpStart REDTaq ReadyMix PCR reaction Mix (Sigma–Aldrich Inc., MO, USA), 1 µl 10 µM forward primer (5’-GAAAATAATTAGTAAATTAGTTAGGTAG-3’), 1 µl 10 µM biotinylated reverse primer (5’- ACTAAAATCACCTCAACAACAAC-3’), 11 µl of PCR grade water, and 100 ng bisulfite-treated DNA. PCR cycling conditions were initial step 96 °C for 5 min, followed by 36 cycles with denaturation at 94 °C for 30 sec, annealing at 55 °C for 45 sec, and elongation at 72 °C for 60 sec, with a final extension at 72 °C for 5 min. Bisulfite pyrosequencing was performed on a PyroMark Q24 pyrosequencer system (Qiagen) with Streptavidin Sepharose Beads (GE Healthcare Bioscience, Uppsala, Sweden), PyroMark Gold Q24 reagents kit (Qiagen), PyroMark workstation buffers (Qiagen), and sequencing primer (5’- GTTATATGATATTTTTAATTT-3’). Data analysis was performed using PyroMark Q24 Software 2.0 (Qiagen) with CpG analysis mode.

### Immunohistochemistry

Immunohistochemistry for SDHA and SDHB proteins was performed as part of clinical assessment of tumors on formalin-fixed, paraffin-embedded tissue sections using AB14715 (Abcam, Cambridge MA, USA) and HPA002868 (Sigma–Aldrich, St. Louis, MO, USA) antibodies, respectively. Briefly, 4 μm thick sections from representative tissue blocks were processed using the Ventana Discovery XT system with antigen retrieval (CC1 solution, 60 min), primary antibody (1:6400 dilution for SDHA (AB14715) and 1:800 dilution for SDHB (HPA002868) antibodies), and OptiView DAB immunohistochemistry detection steps (Ventana Medical Systems, Tucson, AZ, USA).

### Reporting summary

Further information on research design is available in the Nature Research Reporting Summary linked to this article.

## Supplementary information

Supplementary Data Files

REPORTING SUMMARY

## Data Availability

Identifying information for the patients is not available to protect patient privacy. All de-identified tumor DNA sequencing results and associated clinical data for the patients in this study are publicly available in the open-source cBioPortal for Cancer Genomics at https://www.cbioportal.org/study/summary?id=gist_msk_2022.

## Code availability

The MSK-IMPACT data analysis pipeline is available at https://github.com/rhshah/IMPACT-Pipeline. The mutational signature decomposition code is available at https://github.com/mskcc/mutation-signatures.
