# Supplementary material for: Expanded genetic testing of GIST patients identifies high proportion of non-syndromic patients with germline alterations
Source: NPJ Precis Oncol. 2023 Jan 2;7:1. doi: 10.1038/s41698-022-00342-z (PMC9807588; doi:10.1038/s41698-022-00342-z)
Supplement: Supplementary file 1 — Supplementary Data Files [file 41698_2022_342_MOESM1_ESM.docx]

**Supplementary Fig 1: Study cohort overview**

De-identified sequencing data from 499 GIST tumor-normal pairs were analyzed to compare the rate of germline versus somatic P/LP variants in each tested gene. Matched tumor-normal sequencing data was analyzed from 103 patients who consented to germline analysis. These patients received clinical germline genetic testing to identify germline P/LP variants in hereditary cancer predisposition genes, and matched somatic mutation profiling for their tumors, as part of MSK-IMPACT.

**Supplementary Table 1: Genes tested on Germline MSK-IMPACT**

| **GERMLINE MSK-IMPACT 90-GENE VERSION** | **GERMLINE MSK-IMPACT 88-GENE VERSION** | **GERMLINE MSK-IMPACT 76-GENE VERSION** |
| --- | --- | --- |
| *ALK* | *ALK* | *ALK* |
| *APC* | *APC* | *APC* |
| *ATM* | *ATM* | *ATM* |
| *BAP1* | *BAP1* | *BAP1* |
| *BARD1* | *BARD1* | *BARD1* |
| *BLM* | *BLM* | *BLM* |
| *BMPR1A* | *BMPR1A* | *BMPR1A* |
| *BRCA1* | *BRCA1* | *BRCA1* |
| *BRCA2* | *BRCA2* | *BRCA2* |
| *BRIP1* | *BRIP1* | *BRIP1* |
| *CDC73* | *CDC73* | *CDH1* |
| *CDH1* | *CDH1* | *CDK4* |
| *CDK4* | *CDK4* | *CDKN2A* |
| *CDKN2A* | *CDKN2A* | *CHEK2* |
| *CEBPA* | *CEBPA* | *DICER1* |
| *CHEK2* | *CHEK2* | *EGFR* |
| *CTR9* | *DICER1* | *EPCAM* |
| *DICER1* | *EGFR* | *FAM175A* |
| *EGFR* | *EPCAM* | *FH* |
| *EPCAM* | *ERBB2* | *FLCN* |
| *ERBB2* | *ERCC3* | *GATA2* |
| *ERCC3* | *ETV6* | *HRAS* |
| *ETV6* | *FAM175A* | *JAK2* |
| *FANCA* | *FANCA* | *KIT* |
| *FANCC* | *FANCC* | *KRAS* |
| *FH* | *FH* | *MAX* |
| *FLCN* | *FLCN* | *MEN1* |
| *GATA2* | *GATA2* | *MET* |
| *GREM1* | *HOXB13* | *MITF* |
| *HOXB13* | *HRAS* | *MLH1* |
| *HRAS* | *KIT* | *MRE11A* |
| *KIT* | *KRAS* | *MSH2* |
| *KRAS* | *MAX* | *MSH6* |
| *LZTR1* | *MEN1* | *MUTYH* |
| *MAX* | *MET* | *NBN* |
| *MEN1* | *MITF* | *NF1* |
| *MET* | *MLH1* | *NF2* |
| *MITF* | *MRE11A* | *NRAS* |
| *MLH1* | *MSH2* | *PALB2* |
| *MSH2* | *MSH3* | *PAX5* |
| *MSH3* | *MSH6* | *PDGFRA* |
| *MSH6* | *MUTYH* | *PHOX2B* |
| *MUTYH* | *NBN* | *PMS2* |
| *NBN* | *NF1* | *POLE* |
| *NF1* | *NF2* | *PTCH1* |
| *NF2* | *NRAS* | *PTEN* |
| *NRAS* | *NTHL1* | *RAD50* |
| *NTHL1* | *PALB2* | *RAD51* |
| *PALB2* | *PAX5* | *RAD51B* |
| *PAX5* | *PDGFRA* | *RAD51C* |
| *PDGFRA* | *PHOX2B* | *RAD51D* |
| *PHOX2B* | *PMS2* | *RB1* |
| *PMS2* | *POLD1* | *RECQL4* |
| *POLD1* | *POLE* | *RET* |
| *POLE* | *PTCH1* | *RTEL1* |
| *PTCH1* | *PTEN* | *RUNX1* |
| *PTEN* | *RAD50* | *SDHA* |
| *RAD51* | *RAD51* | *SDHAF2* |
| *RAD51B* | *RAD51B* | *SDHB* |
| *RAD51C* | *RAD51C* | *SDHC* |
| *RAD51D* | *RAD51D* | *SDHD* |
| *RB1* | *RB1* | *SMAD3* |
| *RECQL* | *RECQL* | *SMAD4* |
| *REST* | *RECQL4* | *SMARCA4* |
| *RET* | *RET* | *SMARCB1* |
| *RTEL1* | *RTEL1* | *STK11* |
| *RUNX1* | *RUNX1* | *SUFU* |
| *SDHA* | *SDHA* | *TERT* |
| *SDHAF2* | *SDHAF2* | *TGFBR1* |
| *SDHB* | *SDHB* | *TGFBR2* |
| *SDHC* | *SDHC* | *TMEM127* |
| *SDHD* | *SDHD* | *TP53* |
| *SMAD3* | *SMAD3* | *TSC1* |
| *SMAD4* | *SMAD4* | *TSC2* |
| *SMARCA4* | *SMARCA4* | *VHL* |
| *SMARCB1* | *SMARCB1* | *WT1* |
| *SMARCE1* | *STK11* |  |
| *STK11* | *SUFU* |  |
| *SUFU* | *TERT* |  |
| *TERT* | *TGFBR1* |  |
| *TGFBR1* | *TGFBR2* |  |
| *TGFBR2* | *TMEM127* |  |
| *TMEM127* | *TP53* |  |
| *TP53* | *TSC1* |  |
| *TRIP13* | *TSC2* |  |
| *TSC1* | *VHL* |  |
| *TSC2* | *WT1* |  |
| *VHL* | *YAP1* |  |
| *WT1* |  |  |
| *YAP1* |  |  |

**Supplementary Table 2: Characteristics of 103 GIST patients who received germline genetic testing**

| **Group** | **Patient** | **Gender** | **Age at Dx (years)** | **Germline gene** | **Germline variant / classification** | **Somatic driver gene** | **Somatic driver variant** | **Tumor site** | **Focality** | **Tumor type** | **Metastatic*** | **SDH IHC status** | **Syndromic presentation related to identified germline gene** |
| --- | --- | --- | --- | --- | --- | --- | --- | --- | --- | --- | --- | --- | --- |
| **Germline alteration in GIST-associated gene** | 1 | F | 56 | *KIT* | c.1526A>T (p.Lys509Ile) / P | None | N/A | Small Bowel | Multifocal | Spindle | Yes | NA | Yes (Hx or multiple GISTs) |
|  | 2 | F | 60 | *NF1* | c.269T>G (p.Leu90Arg) / LP | None | N/A | Small Bowel | Multifocal | Spindle | No | NA | Yes (Clinical features of NF-1) |
|  | 3 | F | 47 | *NF1* | c.4107C>A (p.Tyr1369*) / P | None | N/A | Small Bowel | Unifocal | Spindle | No | SDHA and SDHB retained | Yes (Clinical features of NF-1) |
|  | 4 | F | 61 | *NF1* | c.8021delC (p.Pro2674Hisfs*44) / P | None | N/A | Unknown | Unknown | Spindle | Yes | NA | Yes (Clinical features of NF-1) |
|  | 5 | M | 53 | *NF1* | c.1381C>T (p.Arg461*) / P | None | N/A | Duodenum | Multifocal | Spindle | No | NA | Yes (Clinical features of NF-1) |
|  | 6 | F | 35 | *NF1* | c.6623_6634delinsCA (p.Trp2208Serfs*33) / P | None | N/A | Small Intestine | Unifocal | Spindle | No | NA | Yes (CAL spots identified after genetic diagnosis) |
|  | 7 | F | 35 | *NF1* | c.339delG (p.Leu113Phefs*52) / P | None | N/A | Small Bowel | Multifocal | Spindle | No | NA | Yes (Clinical features of NF-1) |
|  | 8 | F | 40 | *NF1* | c.2158_2177dup (p.Ser727Glyfs*28) / P | None | N/A | Small Intestine | Multifocal | Mixed | No | NA | Yes (Clinical features of NF-1) |
|  | 9 | F | 56 | *SDHA* | c.1365C>A (p.Asn455Lys) / LP | None | N/A | Stomach | Multifocal | Spindle | Yes | SDHA and SDHB lost | No |
|  | 10 | M | 22 | *SDHA* | c.91C>T (p.Arg31*) / P | None | N/A | Stomach | Multifocal | Mixed | Yes | NA | No |
|  | 11 | F | 19 | *SDHA* | c.1866G>A (p.Trp622*) / LP | None | N/A | Stomach | Multifocal | Mixed | Yes | SDHB lost, SDHA retained | No |
|  | 12 | F | 7 | *SDHA* | c.1753C>T (p.Arg585Trp) / LP | None | N/A | Stomach | Multifocal | Epithelioid | No | SDHB lost, SDHA retained | No |
|  | 13 | M | 21 | *SDHA* | c.91C>T (p.Arg31*) / P | None | N/A | Stomach | Multifocal | Epithelioid | Yes | SDHA and SDHB lost | No |
|  | 14 | F | 71 | *SDHA* | c.1534C>T (p.Arg512*) / P | None | N/A | Stomach | Unifocal | Mixed | Yes | SDHB lost | No |
|  | 15 | M | 27 | *SDHA* | c.91C>T (p.Arg31*) / P | None | N/A | Stomach | Unifocal | Mixed | Yes | SDHA and SDHB lost | No |
|  | 16 | M | 39 | *SDHB* | c.416T>A (p.Leu139His) / LP | None | N/A | Stomach | Unifocal | Mixed | Yes | SDHB lost, SDHA retained | No |
|  | 17 | F | 17 | *SDHB* | c.380T>G (p.Ile127Ser) / P | None | N/A | Stomach | Unifocal | Spindle | No | SDHB lost, SDHA retained | No |
|  | 18 | M | 23 | *SDHB* | c.137G>A (p.Arg46Gln) / P | None | N/A | Stomach | Unifocal | Mixed | Yes | SDHB lost | No |
|  | 19 | F | 37 | *SDHB* | Exon 2 deletion / P | None | N/A | Stomach | Multifocal | Mixed | No | SDHB partially lost, SDHA retained | No |
|  | 20 | M | 57 | *SDHB* | c.287-1G>C (p.?) / P | None | N/A | Stomach | Unifocal | Mixed | Yes | SDHB lost | No |
|  | 21 | F | 54 | *SDHB* | c.268C>T (p.Arg90*) / P | None | N/A | Stomach | Unifocal | Epithelioid | Yes | NA | No |
|  | 22 | F | 51 | *SDHB* | c.380T>G (p.Ile127Ser) / P | None | N/A | Stomach | Unifocal | Mixed | No | SDHB lost, SDHA retained | Yes (Carotid paraganglioma at 27 years of age) |
|  | 23 | F | 54 | *SDHC* | Exon 6 deletion / P | None | N/A | Stomach | Unifocal | Epithelioid | No | SDHB lost, SDHA retained | No |
|  | 24 | M | 18 | *SDHC* | c.380A>G (p.His127Arg) / LP | None | N/A | Stomach | Multifocal | Mixed | No | NA | No |
| **Germline alteration in a gene not known to be associated with GIST** | 25 | M | 66 | *TP53* | c.473G>A (p.Arg158His) / P | *KIT* | c.1669T>A (p.Trp557Arg) | Stomach | Multifocal | Spindle | No | NA | No |
|  | 26 | M | 51 | *MLH1* | c.1852_1854delAAG (p.Lys618del) / P | *KIT* | c.1703_1726delinsGTG (p.Tyr568_Leu576delinsCysVal) | Abdomen | Multifocal | Spindle | Yes | NA | No |
|  | 27 | M | 31 | *BRCA2* | c.7718T>G (p.Leu2573*) / P | *KIT* | c.1655_1668delinsCA (p.Met552_Gln556delinsThr) | Pelvis | Multifocal | Spindle | Yes | NA | No |
|  | 28 | M | 56 | *RECQL* | Exon 11 del / LP | *KIT* | c.1669_1674delTGGAAG (p.Trp557_Lys558del) | Stomach | Unifocal | Epithelioid | Yes | SDHB retained | No |
|  | 29 | M | 79 | *MUTYH* | c.1187G>A (p.Gly396Asp) / P | *KIT* | c.1653_1658delCATGTA (p.Met552_Tyr553del) | Unknown | Unknown | N/A | No | NA | No |
|  | 30 | F | 48 | *MUTYH* | c.463A>T (p.Lys155*) / LP | *KIT* | c.2458G>T (p.Asp820Tyr) | Stomach | Unifocal | Epithelioid | No | SDHA and SDHB retained | No |
|  | 31 | M | 72 | *APC* | c.3920T>A (p.Ile1307Lys) / P | *KIT* | c.1676T>A (p.Val559Asp) | Esophagus | Unifocal | Spindle | No | NA | No |
|  | 32 | M | 58 | *APC* | c.3920T>A (p.Ile1307Lys) / P | *PDGFRA* | c.2525A>T (p.Asp842Val) | Stomach | Unifocal | Mixed | No | NA | No |
| **Somatic mutation in a gene known to be GIST driver** | 33 | M | 62 | None |  | *KIT* | c.1669_1674delTGGAAG (p.Trp557_Lys558del) | Stomach | Multifocal | Spindle | No | NA | No |
|  | 34 | M | 71 | None |  | *KIT* | c.1649_1653delinsTT (p.Lys550_Pro551delinsIle) | Stomach | Unifocal | Spindle | No | NA | No |
|  | 35 | M | 29 | None |  | *KIT* | c.1648_1663delinsT (p.Lys550_Val555delinsLeu) | Stomach | Unifocal | Mixed | Yes | SDHB retained | No |
|  | 36 | F | 66 | None |  | *KIT* | c.1669_1674delTGGAAG (p.Trp557_Lys558del) | Stomach | Unifocal | Epithelioid | Yes | NA | No |
|  | 37 | F | 59 | None |  | *KIT* | c.1648-9_1672del (p.Lys550_Lys558del) | Stomach | Unifocal | Spindle | No | NA | No |
|  | 38 | F | 36 | None |  | *KIT* | c.1679T>A (p.Val560Asp) | Stomach | Unifocal | Spindle | No | NA | No |
|  | 39 | M | 38 | None |  | *KIT* | c.1667_1682delinsC (p.Gln556_Glu561delinsPro) | Stomach | Unifocal | Spindle | No | NA | No |
|  | 40 | F | 59 | None |  | *KIT* | c.1652_1670delinsT (p.Pro551_Trp557delinsLeu) | Stomach | Unifocal | Spindle | No | NA | No |
|  | 41 | F | 52 | None |  | *KIT* | c.1717_1755dup (p.Pro573_Pro585dup) | Stomach | Unifocal | Mixed | No | NA | No |
|  | 42 | M | 58 | None |  | *KIT* | c.1738_1739insCTCCTTATGATC (p.Asp579_His580insProProTrpAsp) | Stomach | Unifocal | Spindle | No | NA | No |
|  | 43 | F | 39 | None |  | *KIT* | c.1725_1774+4dup (p.Gln575_Gly592dup) | Stomach | Unifocal | Spindle | No | NA | No |
|  | 44 | M | 71 | None |  | *KIT* | c.1679T>A (p.Val560Asp) | Stomach | Unifocal | Spindle | No | NA | No |
|  | 45 | M | 58 | None |  | *KIT* | c.1676T>C (p.Val559Asp) | Stomach | Unifocal | Spindle | No | NA | No |
|  | 46 | F | 64 | None |  | *KIT* | c.1655_1660delTGTATG (p.Met552_Glu554delinsLys) | Stomach | Unifocal | Spindle | No | NA | No |
|  | 47 | F | 68 | None |  | *KIT* | c.1671_1674delinsC (p.Trp557_Lys558delinsCys) | Stomach | Unifocal | Spindle | No | NA | No |
|  | 48 | F | 66 | None |  | *KIT* | c.1669_1680delTGGAAGGTTGTT (p.Trp557_Val560del) | Stomach | Multifocal | Spindle | No | NA | No |
|  | 49 | F | 69 | None |  | *KIT* | c.1705_1728del (p.Val569_Leu576del) | Stomach | Unifocal | Spindle | No | NA | No |
|  | 50 | M | 37 | None |  | *KIT* | c.1739_1740insAAATGATTATGATCA (p.Asp579_His580insGlnAsnAspTyrAsp) | Stomach | Unifocal | Spindle | No | NA | No |
|  | 51 | M | 41 | None |  | *KIT* | c.1655_1674delinsGA (p.Met552_Lys558delinsArg) | Stomach | Unifocal | Spindle | No | NA | No |
|  | 52 | M | 47 | None |  | *KIT* | c.1669_1674delTGGAAG (p.Trp557_Lys558del) | Stomach | Multifocal | Mixed | No | NA | No |
|  | 53 | M | 53 | None |  | *KIT* | c.1670_1675delGGAAGG (p.Trp557_Val559delinsPhe) | Unknown | Unifocal | Spindle | Yes | NA | No |
|  | 54 | M | 62 | None |  | *KIT* | c.1676T>A (p.Val559Asp) | Small Intestine | Unifocal | Spindle | No | NA | No |
|  | 55 | M | 36 | None |  | *KIT* | c.1669_1674delTGGAAG (p.Trp557_Lys558del) | Small Intestine | Unifocal | Spindle | No | NA | No |
|  | 56 | M | 50 | None |  | *KIT* | c.1676T>C (p.Val559Ala) | Small Bowel | Multifocal | Epithelioid | Yes | NA | No |
|  | 57 | F | 35 | None |  | *KIT* | c.1665_1679delACAGTGGAAGGTTGT (p.Gln556_Val560del) | Small Bowel | Multifocal | Spindle | Yes | NA | No |
|  | 58 | M | 50 | None |  | *KIT* | c.1727T>C (p.Leu576Pro) | Small Bowel | Unifocal | Mixed | No | NA | No |
|  | 59 | M | 30 | None |  | *KIT* | c.1676T>A (p.Val559Asp) | Small Bowel | Unifocal | Mixed | No | NA | No |
|  | 60 | M | 75 | None |  | *KIT* | c.1727T>C (p.Leu576Pro) | Small Bowel | Multifocal | Mixed | No | NA | No |
|  | 61 | M | 67 | None |  | *KIT* | c.1504_1509dupGCCTAT (p.Ala502_Tyr503dup) | Small bowel | Unifocal | Mixed | No | NA | No |
|  | 62 | F | 58 | None |  | *KIT* | c.2464A>T (p.Asn822Tyr) | Small Bowel | Unifocal | Spindle | No | NA | No |
|  | 63 | M | 63 | None |  | *KIT* | c.1669T>A (p.Trp557Arg) | Small Bowel | Unifocal | Spindle | No | NA | No |
|  | 64 | M | 29 | None |  | *KIT* | c.1684_1728del (p.Glu562_Leu576del) | Small Bowel | Unifocal | Spindle | Yes | NA | No |
|  | 65 | M | 33 | None |  | *KIT* | c.1504_1509dupGCCTAT (p.Ala502_Tyr503dup) | Small Bowel | Unifocal | Spindle | Yes | NA | No |
|  | 66 | F | 24 | None |  | *KIT* | c.1504_1509dupGCCTAT (p.Ala502_Tyr503dup) | Small Bowel | Unifocal | Mixed | Yes | SDHA and SDHB retained | No |
|  | 67 | F | 31 | None |  | *KIT* | c.1665_1727delA (p.Gln556_Leu576del) | Small Bowel | Unifocal | Spindle | No | NA | No |
|  | 68 | F | 42 | None |  | *KIT* | c.1504_1509dupGCCTAT (p.Ala502_Tyr503dup) | Small Bowel | Multifocal | Spindle | Yes | NA | No |
|  | 69 | F | 59 | None |  | *KIT* | c.1657_1674delTATGAAGTACAGTGGAAG (p.Tyr553_Lys558del) | Small Bowel | Unifocal | Spindle | No | NA | No |
|  | 70 | F | 36 | None |  | *KIT* | c.1662_1667delAGTACA (p.Val555_Gln556del) | Small Bowel | Unifocal | Mixed | No | NA | No |
|  | 71 | M | 52 | None |  | *KIT* | c.1961T>C (p.Val654Ala) | Rectum | Unifocal | Epithelioid | No | NA | No |
|  | 72 | F | 60 | None |  | *KIT* | c.1669_1674delTGGAAG (p.Trp557_Lys558del) | Rectum | Multifocal | Spindle | No | NA | No |
|  | 73 | M | 52 | None |  | *KIT* | c.1669_1674delTGGAAG (p.Trp557_Lys558del) | Rectum | Unifocal | Spindle | Yes | NA | No |
|  | 74 | F | 55 | None |  | *KIT* | c.1665_1679delACAGTGGAAGGTTGT (p.Gln556_Val560del) | Rectum | Unifocal | Spindle | No | NA | No |
|  | 75 | F | 63 | None |  | *KIT* | c.1727T>C (p.Leu576Pro) | Pelvis | Unifocal | Spindle | No | NA | No |
|  | 76 | F | 63 | None |  | *KIT* | c.1727T>C (p.Leu576Pro) | Pelvic mass | Multifocal | Mixed | No | NA | No |
|  | 77 | M | 46 | None |  | *KIT* | c.1663_1719del (p.Val555_Pro573del) | Jejunum | Unifocal | Mixed | No | NA | No |
|  | 78 | F | 21 | None |  | *KIT* | c.1705_1728del (p.Val569_Leu576del) | Ileum | Multifocal | Spindle | No | NA | No |
|  | 79 | M | 40 | None |  | *KIT* | c.2466T>A (p.Asn822Lys) | Ileum | Unifocal | Spindle | No | NA | No |
|  | 80 | F | 25 | None |  | *KIT* | c.1504_1509dupGCCTAT (p.Ala502_Tyr503dup) | Duodenum | Unifocal | Spindle | No | NA | No |
|  | 81 | M | 28 | None |  | *KIT* | c.1676T>A (p.Val559Asp) | Duodenum | Unifocal | Spindle | No | NA | No |
|  | 82 | F | 70 | None |  | *KIT* | c.1669T>G (p.Trp557Gly) | Cul-de-sac | Unifocal | Mixed | No | NA | No |
|  | 83 | M | 76 | None |  | *KIT* | c.1504_1509dupGCCTAT (p.Ala502_Tyr503dup) | Abdomen | Unifocal | Epithelioid | No | NA | No |
|  | 84 | M | 51 | None |  | *PDGFRA* | c.2525A>T (p.Asp842Val) | Stomach | Unifocal | Epithelioid | No | NA | No |
|  | 85 | M | 42 | None |  | *PDGFRA* | c.2525A>T (p.Asp842Val) | Stomach | Unifocal | Epithelioid | No | SDHA and SDHB retained | No |
|  | 86 | F | 45 | None |  | *PDGFRA* | c.2525A>T (p.Asp842Val) | Stomach | Unifocal | Spindle | No | NA | No |
|  | 87 | F | 44 | None |  | *PDGFRA* | c.2525A>T (p.Asp842Val) | Stomach | Unifocal | Epithelioid | No | NA | No |
|  | 88 | F | 68 | None |  | *PDGFRA* | c.1682T>A (p.Val561Asp) | Stomach | Unifocal | Epithelioid | No | NA | No |
|  | 89 | F | 57 | None |  | *PDGFRA* | c.2526_2537delCATCATGCATGA (p.Ile843_Asp846del) | Stomach | Unifocal | Epithelioid | No | NA | No |
|  | 90 | F | 63 | None |  | *PDGFRA* | c.2525A>T (p.Asp842Val) | Stomach | Unifocal | Epithelioid | No | NA | No |
|  | 91 | M | 60 | None |  | *PDGFRA* | c.2526_2537delCATCATGCATGA (p.Ile843_Asp846del) | Stomach | Unifocal | Epithelioid | No | NA | No |
|  | 92 | F | 32 | None |  | *PDGFRA* | c.2525A>T (p.Asp842Val) | Stomach | Multifocal | Mixed | Yes | NA | No |
|  | 93 | F | 36 | None |  | *SDHA* | c.1054C>T (p.Arg352*) | Stomach | Unifocal | Spindle | Yes | SDHB lost, SDHA weak | No |
|  | 94 | F | 60 | None |  | *NF1* | Whole gene deletion | Stomach | Unifocal | Spindle | No | NA | No |
|  | 95 | F | 33 | None |  | *NF1* | c.4760delT (p.Leu1587*), c.4935delT (p.Thr1646Profs*52) | Small Bowel | Unifocal | Mixed | Yes | NA | No |
|  | 96 | M | 34 | None |  | *NF1* | c.6852_6855delTTAC (p.Tyr2285Thrfs*5) | Stomach | Unifocal | Spindle | No | NA | No |
|  | 97 | F | 47 | None |  | *NF1* | Exon 9-39 deletion | Duodenum | Unifocal | Spindle | No | NA | No |
|  | 98 | F | 40 | None |  | *BRAF* | AGAP3-BRAF fusion | Bowel | Unifocal | Spindle | No | NA | No |
|  | 99 | M | 42 | None |  | *BRAF* | c.1799T>A (p.Val600Glu) | Duodenum | Unifocal | Spindle | No | NA | No |
| **Somatic mutation status inconclusive** | 100 | F | 60 | None |  | Somatic Inconclusive | Monoallelic SDHB whole gene deletion | Stomach | Unifocal | Epithelioid | No | SDHB lost, SDHA retained | No |
|  | 101 | M | 42 | None |  | Somatic Inconclusive | Monoallelic SDHB whole gene deletion | Duodenum | Unifocal | Spindle | No | NA | No |
|  | 102 | M | 81 | None |  | Somatic Inconclusive | Monoallelic SDHD whole gene deletion | Esophagus | Unifocal | Spindle | No | NA | No |
| **No germline or somatic mutations** | 103 | F | 11 | None |  | None |  | Small Bowel | Unifocal | Epithelioid | No | SDHA and SDHB retained | No |

Dx: Diagnosis; P: Pathogenic, LP: Likely pathogenic,IHC: Immunohistochemistry; F: Female; M: Male; NA: Not available; Hx: History; *Metastasis status at the time of testing

**Supplementary Table 3: Comparison of patients with and without germline P/LP variants in GIST-associated genes**

|  | **Germline P/LP variant in GIST-associated gene**  **(n=24)** | **No P/LP variant in GIST-associated gene**  **(n=79)** | **p-value** |
| --- | --- | --- | --- |
| Median age of onset (years) | 39.5 | 52 | 0.01^a^ |
| Female ratio | 67% | 48% | 0.16^b^ |
| Ratio of patients with multifocal lesions | 50% | 18% | 0.01^b^ |
| Ratio of patients with metastatic disease at the time of testing | 50% | 20% | 0.01^b^ |

Statistical value calculated using Wilcoxon Rank-Sum (a) or Fisher exact test (b).
