# Supplementary material for: Expanded genetic testing of GIST patients identifies high proportion of non-syndromic patients with germline alterations
Source: NPJ Precis Oncol. 2023 Jan 2;7:1. doi: 10.1038/s41698-022-00342-z (PMC9807588; doi:10.1038/s41698-022-00342-z)
Supplement: Supplementary file 2 — REPORTING SUMMARY [file 41698_2022_342_MOESM2_ESM.pdf]

nature portfolio

Corresponding author(s): Ozge Ceyhan-Birsoy and Jorge S. Reis-Filho

Last updated by author(s): Nov 21, 2022

Reporting Summary

Nature Portfolio wishes to improve the reproducibility of the work that we publish. This form provides structure for consistency and transparency in reporting. For further information on Nature Portfolio policies, see our Editorial Policies and the Editorial Policy Checklist.

Statistics

For all statistical analyses, confirm that the following items are present in the figure legend, table legend, main text, or Methods section.

n/a	Confirmed	
☐	☒	The exact sample size (*n*) for each experimental group/condition, given as a discrete number and unit of measurement
☒	☐	A statement on whether measurements were taken from distinct samples or whether the same sample was measured repeatedly
☐	☒	The statistical test(s) used AND whether they are one- or two-sided<br>*Only common tests should be described solely by name; describe more complex techniques in the Methods section.*
☐	☒	A description of all covariates tested
☒	☐	A description of any assumptions or corrections, such as tests of normality and adjustment for multiple comparisons
☐	☒	A full description of the statistical parameters including central tendency (e.g. means) or other basic estimates (e.g. regression coefficient) AND variation (e.g. standard deviation) or associated estimates of uncertainty (e.g. confidence intervals)
☐	☒	For null hypothesis testing, the test statistic (e.g. *F*, *t*, *r*) with confidence intervals, effect sizes, degrees of freedom and *P* value noted<br>*Give P values as exact values whenever suitable.*
☒	☐	For Bayesian analysis, information on the choice of priors and Markov chain Monte Carlo settings
☒	☐	For hierarchical and complex designs, identification of the appropriate level for tests and full reporting of outcomes
☐	☒	Estimates of effect sizes (e.g. Cohen's *d*, Pearson's *r*), indicating how they were calculated

Our web collection on statistics for biologists contains articles on many of the points above.

Software and code

Policy information about availability of computer code

Data collection	Our patient cohort received MSK-IMPACT paired tumor-blood DNA sequencing testing. The MSK-IMPACT data analysis pipeline is available at https://github.com/rhshah/IMPACT-Pipeline. The mutational signature decomposition code is available at https://github.com/mskcc/mutation-signatures.
Data analysis	All de-identified tumor DNA sequencing results and associated clinical data for the patients in this study are publicly available in the open-source cBioPortal for Cancer Genomics at https://www.cbioportal.org/study/summary?id=gist_msk_2022.

For manuscripts utilizing custom algorithms or software that are central to the research but not yet described in published literature, software must be made available to editors and reviewers. We strongly encourage code deposition in a community repository (e.g. GitHub). See the Nature Portfolio guidelines for submitting code & software for further information.

Data

Policy information about availability of data

All manuscripts must include a data availability statement. This statement should provide the following information, where applicable:

- Accession codes, unique identifiers, or web links for publicly available datasets
- A description of any restrictions on data availability
- For clinical datasets or third party data, please ensure that the statement adheres to our policy

Identifying information for the patients is not available to protect patient privacy. All de-identified tumor DNA sequencing results and associated clinical data for the patients in this study are publicly available in the open-source cBioPortal for Cancer Genomics at https://www.cbioportal.org/study/summary?id=gist_msk_2022.

Human research participants

Policy information about studies involving human research participants and Sex and Gender in Research.

Reporting on sex and gender	Sex of the participants have been described in Supplementary Table 2.
Population characteristics	Age at diagnosis, genotypic information and tumor characteristics gave been described in Supplementary Table 2.
Recruitment	Patients were ascertained through their treating physicians and referral was at the discretion of the physicians.
Ethics oversight	All patients provided written informed consent for testing under a Memorial Sloan Kettering Cancer Center Institutional Review Board (IRB)-approved protocol (IRB#12-245).

Note that full information on the approval of the study protocol must also be provided in the manuscript.

Field-specific reporting

Please select the one below that is the best fit for your research. If you are not sure, read the appropriate sections before making your selection.

☒ Life sciences ☐ Behavioural & social sciences ☐ Ecological, evolutionary & environmental sciences

For a reference copy of the document with all sections, see nature.com/documents/nr-reporting-summary-flat.pdf

Life sciences study design

All studies must disclose on these points even when the disclosure is negative.

Sample size	The de-identified cohort consisted of tumor-normal pairs from 499 consecutive patients with GIST who were treated at MSKCC and had MSK-IMPACT. Germline analysis cohort consisted of 103 patients with GIST, who were a subset of the larger cohort and prospectively consented to germline analysis as part of MSK-IMPACT.
Data exclusions	No data was excluded from analysis.
Replication	Tumor-normal sequencing results were analyzed in a paired manner and the matching of both samples from the same patient was confirmed by comparison of the sequencing data.
Randomization	All participants in our study received MSK-IMPACT paired tumor-normal sequencing without randomization.
Blinding	The tumor sequencing results analyses were performed on 499 de-identified tumor-normal pairs and the investigators did not know the identity of the patients. For the germline analysis cohort of 103 patients with GISTs, results were analyzed in an identified manner and de-identification was not possible, because the clinical germline genetic testing results were reported and returned to the patients.

Reporting for specific materials, systems and methods

We require information from authors about some types of materials, experimental systems and methods used in many studies. Here, indicate whether each material, system or method listed is relevant to your study. If you are not sure if a list item applies to your research, read the appropriate section before selecting a response.

Materials & experimental systems

n/a	Involved in the study	
☐	☒	Antibodies
☒	☐	Eukaryotic cell lines
☒	☐	Palaeontology and archaeology
☒	☐	Animals and other organisms
☐	☒	Clinical data
☒	☐	Dual use research of concern

Methods

n/a	Involved in the study	
☒	☐	ChIP-seq
☒	☐	Flow cytometry
☒	☐	MRI-based neuroimaging

Antibodies

Antibodies used

Immunohistochemistry for SDHA and SDHB proteins was performed as part of clinical assessment of tumors on formalin-fixed, paraffin-embedded tissue sections using AB14715 (Abcam, Cambridge MA, USA) and HPA002868 (Sigma-Aldrich, St. Louis, MO, USA) antibodies, respectively.

Validation

AB14715 : https://www.abcam.com/sdha-antibody-2e3gc12fb2ae2-ab14715.html; HPA002868: https://www.sigmaaldrich.com/US/en/product/sigma/hpa002868.

Clinical data

Policy information about clinical studies

All manuscripts should comply with the ICMJE guidelines for publication of clinical research and a completed CONSORT checklist must be included with all submissions.

Clinical trial registration

MSK-IMPACT (ClinicalTrials.gov identifier, NCT01775072)

Study protocol

https://clinicaltrials.gov/ct2/show/NCT01775072

Data collection

The de-identified cohort consisted of tumor-normal pairs from 499 consecutive patients with GIST who were treated at MSKCC and had MSK-IMPACT (ClinicalTrials.gov identifier, NCT01775072) paired tumor-blood DNA sequencing test between April 2015 and June 2021.

Outcomes

To assess whether expanded genetic testing of unselected GIST patients could identify individuals with hereditary predisposition, we analyzed matched tumor-germline sequencing results of MSK-IMPACT from 103 patients with GISTs treated at Memorial Sloan Kettering (MSK) Cancer Center (MSKCC) over a 6-year period. To determine the frequency of somatic versus germline variants identified in tumor-only sequencing of GISTs, we analyzed a cohort of de-identified 499 GISTs that received paired tumor-normal sequencing using MSK-IMPACT, including tumors from the 103 patients in the initial analysis and 396 patients who did not consent to germline testing.
